# Eyecatcher 3.0 – Validating the Use of “Smart Glasses” as a Low-Cost, Portable Method of Assessing Visual Fields

**DOI:** 10.1167/tvst.14.8.7

**Published:** 2025-08-04

**Authors:** Mehal Rathore, Peter F. Reddingius, Peter Campbell, David P. Crabb, Pete R. Jones

**Affiliations:** 1 Department of Optometry and Visual Sciences, School of Health and Medical Sciences, City St. George's, University of London, London, England, UK; 2 Department of Ophthalmology, Guy's and St. Thomas’ NHS Foundation Trust, London, England, UK; 3 UCL Institute of Ophthalmology, London, England, UK

**Keywords:** telemedicine, Smart Glasses, perimetry, glaucoma, home monitoring, visual fields (VFs), feasibility, progression

## Abstract

**Purpose:**

Glaucoma requires regular visual field (VF) assessments. Eyecatcher 3.0 uses novel “smart glasses” hardware to provide a lightweight, low-cost solution, designed for use while unsupervised. This study aimed to determine the feasibility of using Eyecatcher for VF home-monitoring.

**Methods:**

Eyecatcher 3.0 consists of a smartphone, smart glasses, and wireless clicker. Functionally, it attempts to mimic the Humphrey Field Analyzer (HFA; – same task-instructions, stimuli, and outputs, but smaller field of view and luminance range). Five patients with glaucoma used Eyecatcher to test themselves at home for 3 months (both eyes, monocular, once-per-fortnight). Results from a reduced 24-2 grid were compared to HFA data collected in the clinic, and to normative Eyecatcher data collected from 76 normally sighted young adults. A subset of normally sighted participants (*n* = 16) also underwent two additional sessions of follow-up testing to assess repeatability. Usability was assessed via questionnaires.

**Results:**

All Eyecatcher tests were completed successfully (100%). There was reasonable agreement with the HFA in terms of mean deviation (MD; *r* = 0.85, *P* < 0.001) and observed pattern of loss. The HFA exhibited somewhat better repeatability than Eyecatcher (MD Coefficient of Repeatability = 2.9, 95% confidence interval [CI] = 2.1–4.1 decibels [dB] for HFA, vs. 3.9, 95% CI = 2.8–6.1 dB for Eyecatcher), although this difference was not statistically significant. Average Eyecatcher test duration was 6.5 minutes (both eyes). Patients generally rated the Eyecatcher as easy-to-use, although specific concerns were raised by some individuals.

**Conclusions:**

Smart glasses may provide a feasible means of VFs home-monitoring. Eyecatcher yielded similar sensitivity values to the HFA, and most participants found the lightweight smart glasses acceptable to use. Further research is needed to establish diagnostic accuracy and clinical utility.

**Translational Relevance:**

Validation of a new method of glaucoma home monitoring.

## Introduction

Glaucoma is a leading cause of irreversible blindness worldwide, with 112 million cases expected by 2040.^1^ It requires regular, lifelong monitoring.^2^ Every patient should receive a visual field (VF) assessment every 12 months,^3^ with some patients likely benefiting from additional, more frequent testing (e.g. 4 to 8 monthly^4^^–^^8^). These requirements are often unmet, however. Many patients currently wait more than a year between appointments,^9^^–^^11^ leading to instances of avoidable, irreversible sight loss.^12^^,^^13^ The lack of frequent monitoring is particularly concerning for the 3%^4^ to 15%^14^ of individuals with fast-progressing VF loss (i.e. −1.5 decibels [dB]/year or more).

The problem is a global one^15^^–^^19^ and is only likely to increase as societies age. For example, in the United Kingdom (UK), the number of patients with glaucoma are forecast to double in the next 20 years,^20^ despite already accounting for over 1 million UK hospital outpatient appointments per year^21^ (20% of the total ophthalmic workload).

Telemedicine, and, in particular, vision home-monitoring, has been suggested as a potential solution to the twin challenges of oversubscribed clinics and insufficient monitoring of high-need cases.^22^ Its proponents suggest variously that it might allow hospital appointments to be shortened (by collecting VF data in advance), to be reduced in frequency for low-risk patients, to be conducted remotely, or for additional “high-frequency” VF testing to be performed in newly referred or high-risk individuals. In addition, whereas telemedicine has historically been of niche interest in ophthalmology (although see Refs. 23–25), interest has surged, post-coronavirus disease (COVID), both within glaucoma,^22^^,^^26^ and across other chronic eye conditions.^27^^,^^28^

Recently, we conducted a 6-month pilot of vision home-monitoring, using a tablet-based threshold perimeter (Eyecatcher 2.0).^29^ Consistent with others,^30^ we observed good association between VFs measured at home and in the clinic (MD correlation: *r* = 0.94, *P* < 0.001), and good adherence (98%). However, conversations with patients also raised practical concerns,^31^ including (i) the cost of the equipment; (ii) the size of the equipment (which patients found heavy and inconvenient to store/transport); (iii) a lack of control over viewing distance and ambient lighting; (iv) difficulties remembering to patch the fellow eye; and (v) difficulties connecting to the internet to upload/transmit data.

**Figure 1.**
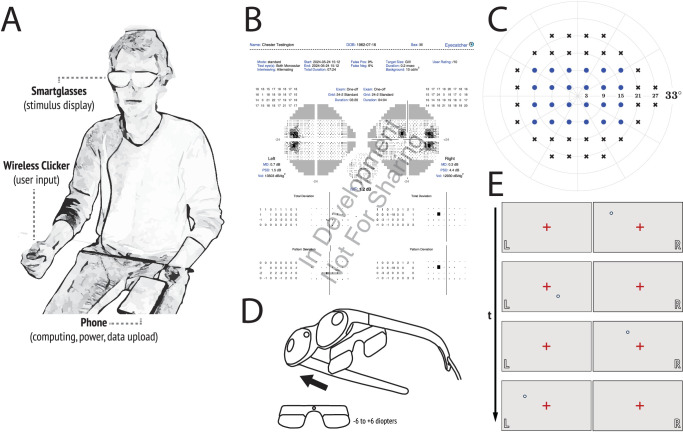
Eyecatcher 3.0. (**A**) Hardware: Eyecatcher ran on an Android smartphone connected to a wireless clicker and a pair of XReal Light smart glasses. (**B**) Output: an example .pdf printout from the Eyecatcher device, showing the data from both eyes together. (**C**) Test grid: the subset of the 24-2 grid assessed. (**D**) Optics: magnetic lens inserts were used to correct for spherical error, as required. (**E**) Test eye interleaving: the target was presented alternately to each eye.

To address these practical challenges, we comprehensively redesigned the Eyecatcher visual field test to run on “smart glasses” (see Fig. 1). Unlike a conventional virtual reality headset, smart glasses consist of relatively ordinary-looking glasses with two integrated display screens (one per eye). They are often relatively lightweight (70–200 g) and inexpensive (US $200 to $500) – owing to the fact that they rely on a connected smartphone/computer to provide all of their power and data-processing. There is no need for patching because stimuli can be presented independently to either eye. In addition, because the screens are effectively “fixed” to the patient's head, viewing distance and ambient lighting can be controlled precisely.

In this initial study, we assessed the feasibility of using novel smart glasses technology as a portable static threshold perimeter (Eyecatcher 3.0), by asking 5 patients with glaucoma to test themselves at home for 3 months and, for comparison, assessing 76 normally sighted young adults in the clinic.

## Methods

### Participants

Participants were 5 patients with glaucoma and 76 normally sighted young adults. As detailed in the Table, the 5 glaucoma patients were aged 67 to 75 years (mean = 73 years), and all had an established diagnosis of stable glaucoma (MD loss/year < 2 dB, as confirmed by at least 4 years of Humphrey Field Analyzer [HFA] data). The ability to perform standard automated perimetry (SAP) reliably was not an inclusion criterion. However, as shown in the Table, all patients did, in practice, show low false positive/negative response rates.

**Table.** Glaucoma Patient Clinical Characteristics

Glaucoma Patients’ Characteristics										
					Mean HFA MD, dB					
			Optical Prescription, Mean Spherical Error		Visit 1 (pre)		Visit 2 (Post)			
Study ID	Age, Y	Diagnosis	OS	OD	OS	OD	OS	OD	FP%	FN%
003	69	PACG	−0.50	−0.63	−26.3	−5.9	−27.1	−5.2	1.6	3.6
004	75	POAG	−2.00	−0.50	−3.0	−0.1	−4.4	−2.4	7.9	8.5
010	75	POAG	−6.50	−5.00	−13.1	−11.4	−11.4	−10.4	1	1.8
014	70	POAG	0.25	−0.25	−8.5	−20.3			0.8	3.8
017	72	POAG	−0.75	−1.00	−10.7	−24.0	−9.6	−22.9	1.1	1.9

PACG, primary angle closure glaucoma; POAG, primary open angle glaucoma.

As described in the Results section, participant 014 did not complete the final HFA evaluation because of an injury that prevented them from attending their final appointment.

**Figure 2.**
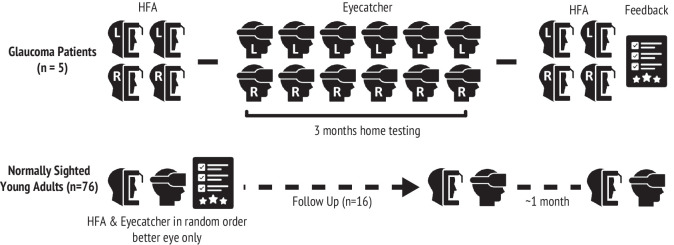
Testing protocol. Patients performed 3 months of home testing, with in-clinic HFA assessments before and after. Both eyes were tested monocularly. Normally sighted healthy controls were tested in the clinic only, with only one eye assessed once – although a subset of 16 healthy controls also repeated the test twice at a later date, to assess test-retest repeatability. These retests were performed in separate sessions, the first occurring around 2 months after the main study, and the second 1 month later.

HFA MD, dB indicates the mean MD value from two HFA tests (see Figure 2 for protocol). FP% and FN% indicate the false positive and false negative values, averaged across all HFA assessments for both eyes (*n* = 8 for all but participant 014).

The 76 normally sighted participants were aged between 18 and 30 years (mean = 21 years) and were recruited from a university research database. Normal vision was defined as: (i) monocular visual acuity ≤ 0.2 logMAR (as measured at 3 m using an Early Treatment Diabetic Retinopathy Study [ETDRS] letter chart; Precision Vision, Woodstock, IL, USA); (ii) monocular contrast sensitivity ≥ 1.50 logCS (as measured at 1 m using the Pelli-Robson chart; Precision Vision, Woodstock, IL, USA); (iii) VF within normal limits (as measured using the HFA, running 24-2 SITA Fast; Carl Zeiss Meditec Ltd., Dublin, CA, USA); and (iv) no self-reported eye conditions. Two additional people were screened but were excluded from the study (*n* = 78 screened).

The study was approved by the Ethics Committee of City, University of London (#ETH2021-2265/2105) and adhered to the tenets of the Declaration of Helsinki. Patients with glaucoma were entitled to travel expenses. Normally sighted participants received a £10/hour compensation for their time.

### Overview of the Novel Eyecatcher Device

As shown in Figure 1A, Eyecatcher 3.0 is a head-mounted perimeter incorporating inexpensive “XReal Light” smart glasses (XReal Inc., Beijing, China). It is being developed by Irida Health Ltd. (a spin-out from City, University of London), but is not yet commercially available.

As per conventional SAP, the user is asked to fixate a central light, and press a button when a target (Goldmann III spot; ⌀ = 0.43 degrees) of varying luminance and spatial-location is seen. Target intensity is varied trial-by-trial using a custom maximum likelihood algorithm. The exact details of this algorithm are proprietary, but it essentially consists of a modified QUEST+ procedure,^32^ which is, in turn, a more sophisticated/generalized superset of the familiar ZEST perimetric algorithm.^33^ As shown in Figure 1B, the output is similar to that of the HFA, and includes patient details, reliability indices, greyscales, total/pattern deviation plots, and various summary metrics including mean deviation (MD). False positive and false negative response rates were assessed using explicit minimum-/maximum-luminance stimuli (i.e. as per historic SAP, before these trials were removed in commercial perimeters to save time^34^).

In this study, the background luminance was fixed at 10 cd/m^2^ and targets were 200 msec Goldmann III white circles, arranged on a reduced 24-2 test grid (see Fig. 1C). Bitstealing^35^ was used to achieve greater-than-10-bit luminance precision, and the luminance of each pixel of every display was individually calibrated using a custom-made robotic optical system designed for imaging inside head-mounted displays. Note that whereas smart glasses typically allow the external environment to remain visible (i.e. with the screen outputs superimposed onto the outside world as a “head up display”), for our purposes, we fitted a light-weight plastic occluder to the front of the glasses. This blocked all incoming light from the front, and partially prevented light ingress from the sides also, allowing the adapting luminance of 10 cd/m^2^ to be maintained. Magnetic insert lenses (Fig. 1D) were used as required to correct for refractive error (at 2 m – the projected distance of the images on the screen). The magnetic frames housing the lenses were manufactured by XReal (XReal Inc., Beijing, China), whereas the lenses themselves were manufactured by Essilor (Essilor International, Paris, France). These lenses were used to correct for Spherical Equivalent (SE) refractive error, in 0.5 diopter steps from –8 to +8 diopters. Outside of this range, the users would be required to wear contact lenses, similar to the HFA, although in practice this did not occur during the study.

During the test, verbal encouragement and instruction were provided by a speech synthesizer, via speakers embedded in the glasses. This encouragement consisted primarily of generic phrases (“keep it up,” and “doing well”) selected randomly at random intervals (constrained to occur no more than once per minute), as well as important temporal information (e.g. “test starting in 3..2..1..,” and “about 1 more minute to go”). Additional feedback based on the pattern of user responses (e.g. if no button was pressed for over a minute, or other anomalous behaviors were detected) was also available, but was seldom, if ever, triggered.

With Eyecatcher, either one eye can be tested monocularly (as per the HFA), both eyes tested monocularly (interleaved), or both eyes tested binocularly. In the present study, controls were tested monocularly (single eye only), whereas both eye monocular testing was used in patients, with the test eye alternating trial-by-trial (Fig. 1E). Note that on any single trial, the user is unaware of which eye the target was presented to, obviating any psychological effects from knowing that the better/worse eye is currently being assessed. No patching was used, as stimuli were presented independently to each eye, with negligible light leakage between the two.

Target ΔLuminance (increment from background) varied from 0.1 to 140 cd/m^2^ (i.e. 10.1 to 150 cd/m^2^ in absolute luminance). This corresponds to 13.6 to 45 dB on the HFA decibel scale, and, for ease of comparison, all data in the present manuscript are reported in the HFA dB scale. Note that this means that due to the limited dynamic range of the smart glasses, very intense stimuli could not be presented (NB: for comparison, the maximum intensity of the HFA is 3183 cd/m^2^).

The gamma function was approximately 2.2. However, note that, in accordance with best practices (and to account for local irregularities in the input-output function), we did not use a gamma function to calibrate the luminance output of the glasses. Instead, a brute force approach was used in which the luminance output for every possible input level was measured empirically, for every pixel of each display of every headset. This calibration was performed using a custom-built robotic test rig, which was itself calibrated using an integrating sphere with in-built photometer (Labsphere Inc.; North Sutton, NH, USA).

In addition, note that given the limited field of view (FoV) of the glasses (W × H = 45 degrees × 25.5 degrees) the most peripheral of the 30 locations on the 24-2 grid were not presented, and testing was restricted to the reduced 24-2 grid shown in Figure 1C. This is a significant limitation of the hardware, although one that may reduce over time as newer screen technologies are developed (see Discussion).

Finally, note that the screens within the smart glasses screen are flat, unlike the conventional SAP bowl (a “tangent perimeter”). The target stimuli were therefore warped in software to be invariant in size and shape across the VF. However, this correction was applied only for mathematical completeness. Given the limited FoV, the required changes to the stimuli were minimal, and it is unlikely that the results would be measurably affected if this correction had not been applied.

Unlike newer models released subsequently, the XReal smart glasses used in the present study assumed a fixed interpupillary distance (IPD) of 63 mm (with an anecdotally reported tolerance of approximately ± 8 mm). Because IPD could not be adjusted, it was not measured for participants, and to the extent that participants differed in their true IPD, this may have contributed to random or systematic measurement error. A crude workaround was implemented in which patients could manually adjust the horizontal placement of the fixation cross. However, in practice, this feature was not used, as no participants reported, when questioned, any difficulty fusing the fixation cross. Newer smart glasses allow IPD to be adjusted to each user, although this customization would add some additional time to the overall test duration.

### Study Protocol

#### Overview

As shown in Figure 2, the 5 patients with glaucoma were each given an Eyecatcher device (see Supplementary Fig. S1 for the hardware and case provided) to take home for 3 months and were asked to test both eyes, monocularly, once every 2 weeks (6 tests total per eye; and 12 tests total per patient). No reminders were issued, although in practice all patients successfully completed the regimen. Patients were given a short, informal demonstration of the device in the clinic, but all testing was performed at home, unsupervised. Meanwhile, the 76 normally sighted participants were tested only once, in the clinic (better eye only, as determined by visual acuity). A subset of 16 healthy controls were also invited for a follow-up in which 2 further Eyecatcher tests were performed, 1 month apart, in order to assess test-retest variability.

#### Humphrey Field Analyzer Reference Measures

Reference measurements were made with the HFA (Carl Zeiss Meditec, Dublin, CA, USA), using a 24-2 grid, the SITA Fast^36^ algorithm, and the same background level and stimulus size/duration as Eyecatcher (10 cd/m^2^ background, 200 msec Goldmann III targets). SITA Fast was used rather than SITA Standard, as SITA Fast exhibits only marginally lower reliability,^34^ and has become the de facto standard in many clinics.

As shown in Figure 2, patients performed two HFAs in each eye, directly before and after the home monitoring period (4 HFAs per eye, total). In the healthy controls, HFA measurements were made on the same day as Eyecatcher, with the order of Eyecatcher and HFA randomly counterbalanced between participants.

#### Usability Feedback

At the end of the study, the five patients with glaucoma were asked to provide feedback regarding the usability of Eyecatcher via the System Usability Scale (SUS).^37^ The SUS is a well-established, general-purpose, Likert-based questionnaire for assessing the perceived usability of digital technologies, comprised of 10 statements including “I felt very confident using the system” and “I think I would need the support of a technical person” (see Supplementary Table S1 for the full listing).

The 76 healthy controls were also asked about usability, but due to time constraints they were not given the SUS, and were instead given 5 Likert statements (“I found the test... enjoyable, easy to perform, tiring, hard to concentrate on,” and “I understood the test well”).

## Results

### Completion Rates

The Eyecatcher completion rate was 100% for all participants (including for the 60 Eyecatcher tests performed at home by people with glaucoma, unsupervised), with no adverse events encountered. Notably, the HFA completion rate was 99% (across all patients and controls) as one patient was unable to attend their final appointment due to injuries sustained in a minor road accident. (The Eyecatcher test equipment was instead mailed back, and the patient debriefed over the phone.)

### Accuracy (Agreement With Reference Measures)

**Figure 3.**
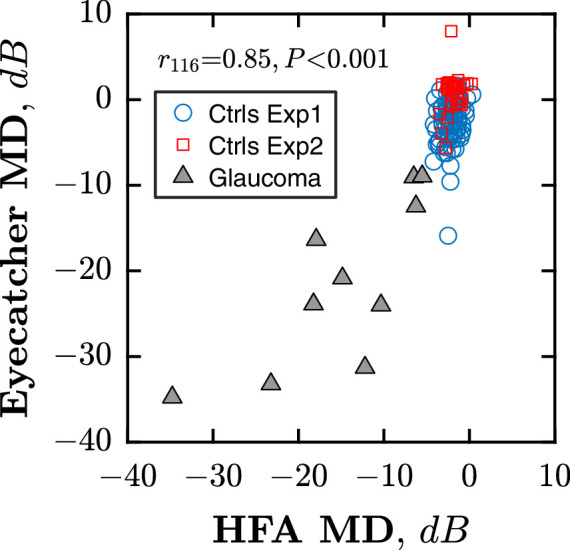
Scatterplot showing Pearson correlation between Eyecatcher and HFA, in terms of mean deviation (MD). Markers represent mean MD values for individual eyes (*grey triangles* = 5 patients with glaucoma tested at home; *blue circles* = 76 controls; and *red squares* = 16 follow-ups). Note that the raw Eyecatcher values were rescaled to be in the HFA dB scaled, as detailed in the Methods section.

As shown in Figure 3, Eyecatcher and HFA MD values were significantly correlated (Pearson's Correlation = *r_116_* = 0.85, *P* < 0.001), with 80% of MD values differing by 3.6 dB or less. The correlation was *r_8_* = 0.78, *P* = 0.008 if just considering the 10 glaucomatous eyes. Across all eyes, the intraclass correlation coefficient (ICC) 95% confidence interval (95% CI) was *r* = 0.78, 95% CI = 0.70–0.84.

**Figure 4.**
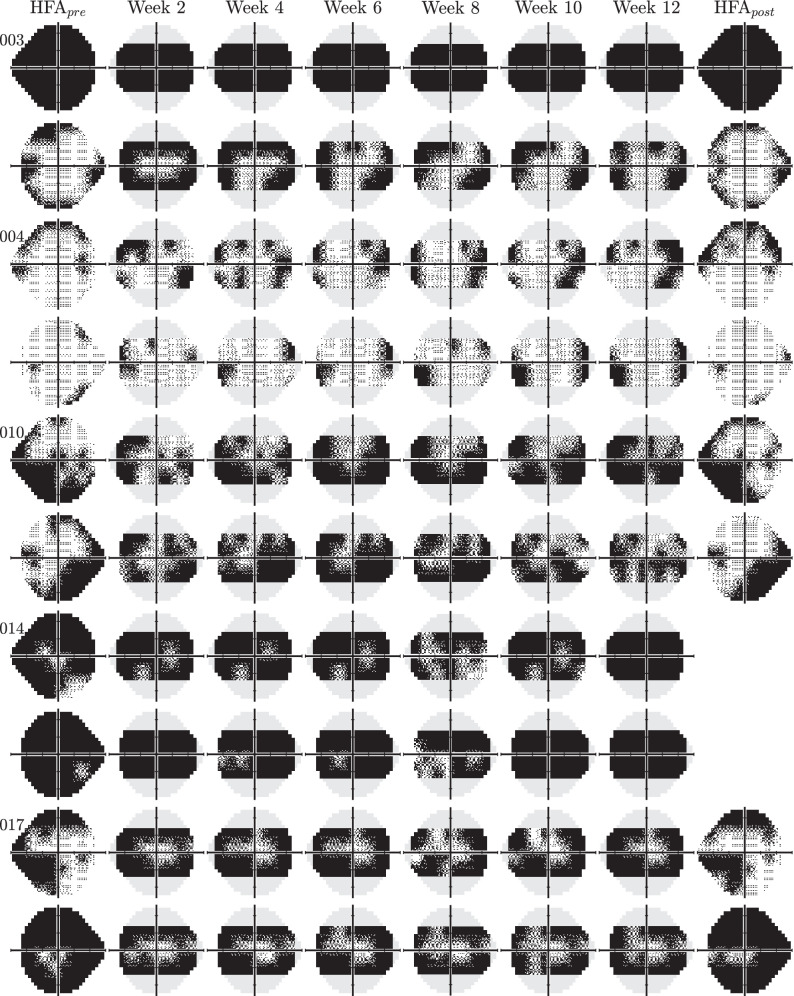
All of the visual field greyscales generated by patients with glaucoma (n = 10 eyes). Each individual was asked to perform the Eyecatcher tests once every 2 weeks, at home, unsupervised (6 tests total per eye). The *first* and *last columns* show the HFA data, before and after the Eyecatcher test period (mean of 2 tests performed same day). Participant #014 was unable to attend his final HFA assessment due to an injury (see main text). Note that for ease of comparison, the HFA greyscales have been “thresholded,” such that all pointwise values lower than 13.6 dB (i.e. outside of the Eyecatcher's dynamic range) have been set to 0. The raw, unadulterated data can be seen in Supplementary Figure S2.

At the pointwise level, 80% of individual points differed by 6.3 dB or less (median absolute difference = 2.8 dB), and concordance in the pattern of loss is evident by inspection of the raw greyscales (see Fig. 4). Although imperfections can also be observed (e.g. the exaggerated inferior field loss in patient 004’s right eye, or the inferotemporal defect in patient 017’s right eye). Some eyes also appeared to exhibit a somewhat more constricted field versus the HFA (e.g. patient 003 OS and patient 017). In part, that may be an artifact of the smoothing that is applied when producing the greyscales, although we cannot rule out other causes, including perceptual effects (e.g. rim artifacts from the lens inserts), cognitive/attentional effects (for points falling close to the visible boundary of the display), or calibration errors in the far periphery. In addition, evident in Figure 4 is the reduced spatial extent of the Eyecatcher visual field (due to limitations of the present hardware).

As would be expected, MD significantly differed between tests from patients with glaucoma and healthy controls (t_198_ = 22.85, *P* < 0.001), and there was near-perfect separation between eyes with mild/no visual field loss and those with pronounced visual filed loss – with an Area Under the Receiver Operating Curve (AUROC) { of 0.99, 95% CI = 0.95 to 1.00, AUROC = 0.99, 95% CI = 0.96 to 1.00, and AUROC = 1.00, 95% CI = 1.00 to 1.00, when separating eyes with mild/no VF loss (MD_HFA_ > –5 dB) from those with moderate (MD_HFA_ = –5.01 to –11.99 dB), advanced (MD_HFA_ = –12.00 to –19.99 dB), or severe (MD_HFA_ ≤ –20 dB) VF loss, respectively. This should not, however, be taken to indicate that Eyecatcher is perfectly capable of detecting glaucoma. Careful inspection of Figure 3, for example, shows a relative absence of patients with mild/early visual field loss in our sample, and even in our cohort of patients with mid-to-late stage glaucoma 3% of the healthy controls exhibited a poorer MD than the best performing patient (MD = –7.2 dB).

### Reliability (Test-Retest Repeatability)

**Figure 5.**
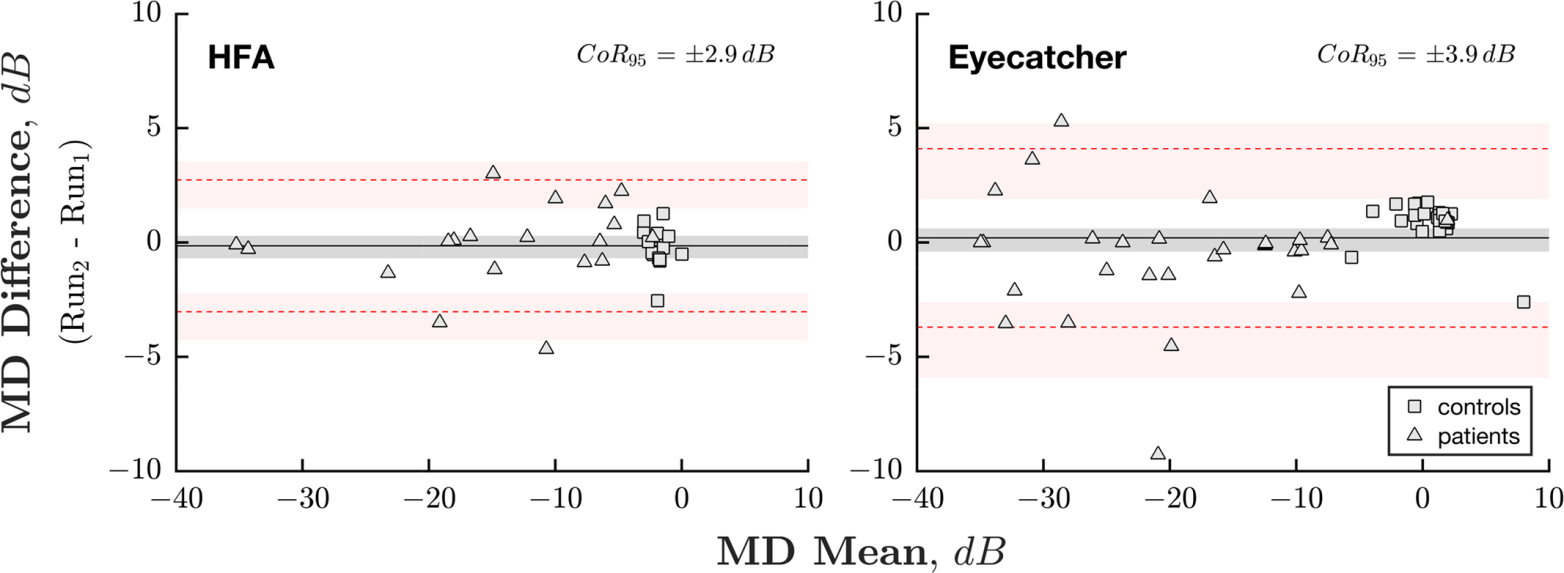
Bland-Altman plots depicting the test-retest repeatability of the HFA (*left*) and the Eyecatcher (*right*). Markers represent individual eyes from controls (*squares*; *n*_HFA_ = 16, *n*_Eyc_ = 32) and patients (*triangles*; *n*_HFA_ = 18, *n*_Eyc_ = 30). The *solid horizontal lines* show the bias (mean difference [MD]) in MD values. The *dashed horizontal lines* show the 95% limits of agreement (1.96 standard deviations [SDs] from the mean). The *shaded regions* denote 95% confidence intervals, derived using bootstrapping (*N* = 20,000; bias corrected and accelerated method). Note that for the Eyecatcher, when analyzing the patients, differences were computed between each successive pair of home tests.

Figure 5 shows test-retest repeatability (of MD) for both Eyecatcher and the HFA, as measured in patients with glaucoma and the 16 follow-up healthy controls. The 95% CI was smaller for the HFA (2.9, 95% CI = 2.1–4.1 dB) than Eyecatcher (3.9, 95% CI = 2.8 – 3.1 dB), indicating that the HFA produced somewhat more reliable, consistent data. However, Eyecatcher's repeatability value fell within the HFA's 95% CI, meaning that this difference was not statistically significant (*P* > 0.05).

Perimetry often exhibits a learning effect. Consistent with this, a small but significant Eyecatcher change in MD was observed for controls (*P* < 0.001), although not for patients (*P* = 0.086). See Supplementary Figure S3 for details.

### Test Durations

**Figure 6.**
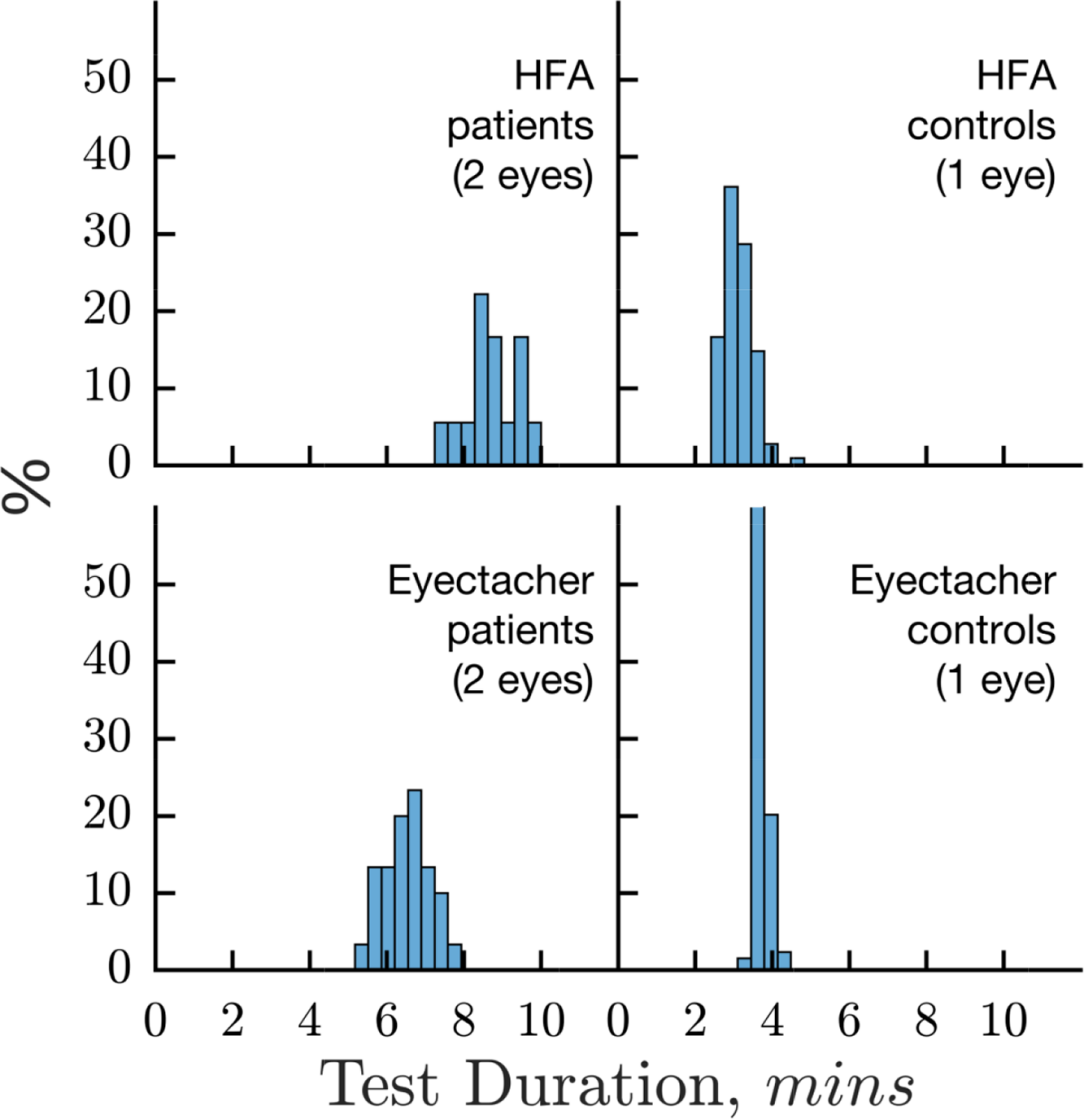
Histograms showing the distribution of test duration for the Eyecatcher and the HFA. Note that patients tested both eyes (monocularly), and the combined time for both eyes is given. Controls only tested one eye. In total, patients completed 18 pairs of HFA assessments and 60 pairs of the Eyecatcher assessments, whereas healthy controls completed 76 single-eye HFA assessments and 152 single-eye Eyecatcher assessments.

For patients with glaucoma, the median (quartiles) total time taken to test both eyes was 8.9, quartiles = 8.4 to 9.6 minutes for the HFA, and 6.5, 95% CI = 6.2–6.9 minutes for Eyecatcher (Fig. 6). This difference was significant (Mann-Whitney *U* test; *Z* = 6.33; *P* < 0.001), even without taking into account the additional time required by the HFA in order to physically swap over the test eye (an additional 1–2 minutes on average), or the fact that Eyecatcher included false-negative (“easy”) catch trials. However, this is to be expected, given that the HFA test grid included almost twice as many test locations (32 vs. 54). In contrast, in healthy controls, the median (quartiles) test duration was actually faster (*Z* = –11.76, *P* < 0.001) for the HFA (3.1, quartiles = 2.8–3.3 minutes) than Eyecatcher (3.7, quartiles = 3.6–3.8 minutes), even despite the additional test locations, indicating that SITA Fast is remarkably well optimized for normally sighted eyes. Note, however, that this difference of approximately 40 seconds is substantially smaller, for example, than the time that was required to seat the control participants and explain the HFA test procedure in the present study (median = 141 seconds, interquartile range [IQR] = 155 seconds), suggesting that the difference in test duration may be relatively small in practical terms. Overall, these data indicate that the two tests are broadly similar in speed.

### Usability

The median SUS score for patients with glaucoma was 93 of 100, with individual patients giving SUS scores of 93, 100, 93, 45, and 68. For context, a score above 80 is generally considered an “A” (very easy to use), whereas a score below 50 is an “F” (very hard to use). Thus, the data indicate that most but not all patients found Eyecatcher very easy to use. A complete breakdown of answers is given in Supplementary Table S1. All patients reported that they would not need technical assistance to operate the system and that most people would learn to use it quickly, but some patients found visual field testing cumbersome and would not like to perform it frequently.

Most of the young-adult normally sighted group reported that they understood the test well (82.9%) and that it was easy to perform (59.2%) and enjoyable (59.2%). Although 31 (40.1%) and 28 (36.8%) people stated that the test was hard to concentrate on and tiring, respectively (see Supplementary Fig. S4). Note that due to an oversight, equivalent ratings were not elicited for the HFA, so no direct comparison between the devices can be made.

## Discussion

This study demonstrates the feasibility of using a portable, smart glasses-based perimeter (Eyecatcher 3.0) to assess glaucomatous visual fields at home, unsupervised (“telemedicine”).

### On the Use of Smart Glasses

Head mounted displays (HMDs) confer several benefits over conventional bowl perimeters (e.g. control over ambient lighting and viewing distance, no need for patching, reduced size and weight, easier transport, and better accessibility). In addition, several previous groups have investigated the use of HMDs for assessing VFs.^38^^–^^44^ However, the present study is one of the first in which patients have used such a device at home, unsupervised,^45^^,^^46^ and the first to use low-cost smart glasses as opposed to conventional virtual reality displays.

Because the smart glasses draw their power and computing over USB from an attached device (smartphone/computer), they are substantially cheaper and lighter than conventional virtual reality headsets. For instance, the smart glasses used in the present study retailed for US $399 at the time of writing (similar to the cost of 1-to-2 years’ worth of topical glaucoma medication in many countries^47^^–^^49^), and weighed approximately 125g – over 3 times lighter than, for example, the Meta Quest 3 (Meta, Menlo Park, CA, USA; 513g) and other similar HMDs used previously by other groups.^50^^–^^52^ We have found, in particular, that bulkier HMDs are often not tolerated by older, frailer patients (indeed, following this study, the Eyecatcher headset was further reduced to < 75g based on patient feedback). The Eyecatcher software also benefits from other features that set it apart from other devices, including individual-pixel luminance calibration, 10-bit luminance control, refractive correction via lens inserts, 5G cloud synchronization, DICOM connectivity with electronic medical records, and compatibility with the Open Perimetry Interface.^53^

### Agreement With Previous Literature

In terms of test performance, the level of association for global measures of sensitivity observed between the HFA and Eyecatcher (*r* = 0.83) was similar to that reported for other portable perimeters, such as the Melbourne Rapid Fields (*r* = 0.85)^30^ and Olleyes VisuALL (*r* = 0.5–0.8 depending on the participants).^40^ Agreement was lower, however, than reported previously, using an earlier, tablet-based iteration of Eyecatcher (*r* = 0.94).^29^ This change was not entirely unexpected. In part, it may reflect a change to new, faster algorithms (test durations were approximately 50% faster in the present study). It may also reflect the transition from tried-and-tested hardware, to an entirely new form of displays (waveguided smart glasses’ panels) that presents substantive new challenges in terms of stimulus calibration and head positioning. Eyecatcher remains in active development, and changes to the device have already taken place following the present study (see below).

In terms of glaucoma vision home-monitoring in general, the present study indicated that at least some patients are able and willing to perform VF testing at home (and notably, one was able to do so even when too injured to attend in-person for an HFA assessment). The adherence rate of 100% reported in the present study is marginally higher than the 97% to 98% reported by ourselves and others previously.^29^^,^^30^ However, all of these numbers likely overestimate how telemedicine would really fare in routine clinical practice (i.e. owing to the highly selective nature of research volunteers). Pending a formal randomized clinical trial (RCT) of ophthalmic telemedicine, we believe that somewhat more realistic insight can be found in Bianchi et al.,^54^ where 233 random patients with cataract were mailed a pen-and-paper test to complete at home, to which 108 patients (46%) responded. A small Eyecatcher learning effect was observed in the healthy controls, and this too is broadly consistent with previous data.^55^^,^^56^

### Limitations and Future Work

Regarding Eyecatcher itself, the device is limited by the small size of the screen (precluding assessment of the full 24-2 grid), and the limited luminance range (precluding quantification of very deep scotomas). These limitations will likely be mitigated by continuing, incremental improvements to commercial hardware (e.g. since this study was performed, newer smart glasses have become available with triple the maximum luminance output – allowing testing down to approximately 8 dB_HFA_). The present study also demonstrates that the software could be improved, given that the SITA Fast test algorithm was faster in normally sighted controls and produced more repeatable results.

Another important practical consideration is that, in the present study, each pixel of each display was individually calibrated. Whether this is strictly required, or whether a single “exemplar” calibration would be sufficient for every headset of the same make and model remains an open question (further data collection and analysis are ongoing). However, preliminary observations seem to indicate substantial device heterogeneity, with luminance output differences of up to 25% observed between different pixels/displays (as well as occasional “dead pixels” or other abnormalities). This raises significant quality control considerations, and would mean in practice that users wishing to precisely control the luminance of the target and/or background could not, for example, simply download the Eyecatcher software onto their own uncalibrated device and would instead need to be supplied with calibrated hardware or follow some defined calibration protocol.

Regarding the present study, the key limitation is the size and self-selecting nature of the patient sample. A larger sample is also required to quantitatively evaluate the pointwise concordance between Eyecatcher and the HFA (data collection ongoing). Furthermore, the present study is only intended as a feasibility pilot, and a much larger study examining a representative cross-section of patients is required to formally assess diagnostic accuracy or clinical utility (see above). Thus, whereas the present study indicates that portable smart glasses can be used to remotely monitor VFs in some patients, it should not be taken as evidence that such technology would scale effectively to routine clinical practice. The present study was also not designed to assess the clinicoeconomic utility of portable VF technologies, and it remains to be seen exactly what the best clinical use cases for them are (e.g. home-monitoring, screening, or alternative care pathway for individuals unable to attend in-person clinics). Furthermore, the present study does not include patients with early-stage glaucoma, or any age similar healthy controls (data collection for which is ongoing). It does not therefore provide any information regarding, for example, Eyecatcher's ability or inability to detect early glaucoma.

### Conclusions

This study indicates that a novel, smart glasses based perimeter (Eyecatcher 3.0) is capable of monitoring the vision of some patients with glaucoma at home, widening the scope for telemedicine in eye care. Although there are several practical advantages of using these glasses over conventional perimeters, technical aspects like screen size and luminance range would benefit from further refinement. Further research is also required to formally establish diagnostic accuracy for detecting progression, and to determine the most appropriate use for portable/home perimetry technologies.

## Supplementary Material

Supplement 1
