# Supplementary material for: Eyecatcher 3.0 – Validating the Use of “Smart Glasses” as a Low-Cost, Portable Method of Assessing Visual Fields
Source: Transl Vis Sci Technol. 2025 Aug 4;14(8):7. doi: 10.1167/tvst.14.8.7 (PMC12327542; doi:10.1167/tvst.14.8.7)
Supplement: Supplement 1 [file tvst-14-8-7_s001.pdf]

SUPPLEMENTAL MATERIAL

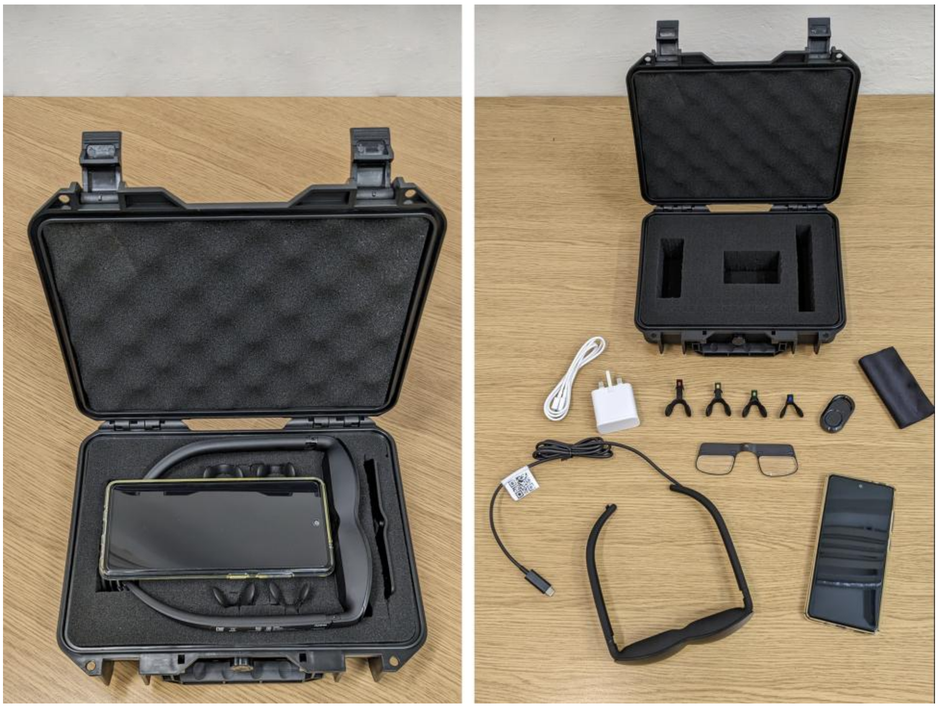

Supplemental Figure S1: Boxed version of Eyecatcher, as supplied to patients undergoing home monitoring. Including smartphone, smartglasses, Bluetooth response button, cleaning cloth, nose pieces, and magnetic lens inserts.

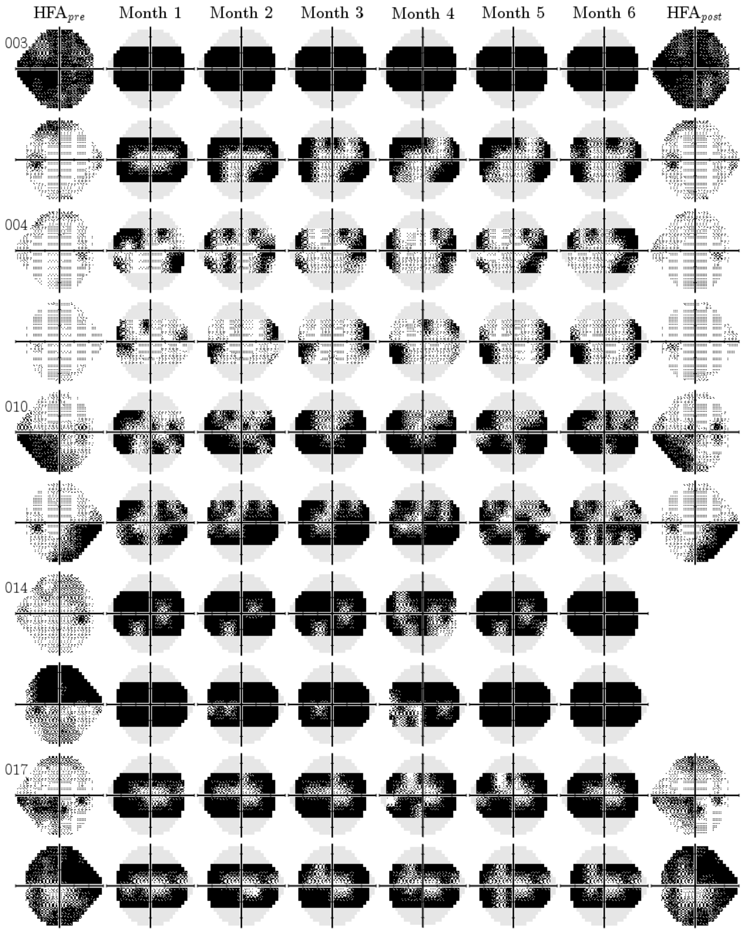

Supplemental Figure S2: Alternative version of **Figure 4** (main manuscript), showing the raw, unadulterated HFA data (fist/last columns) without thresholding for equivalence with Eyecatcher.

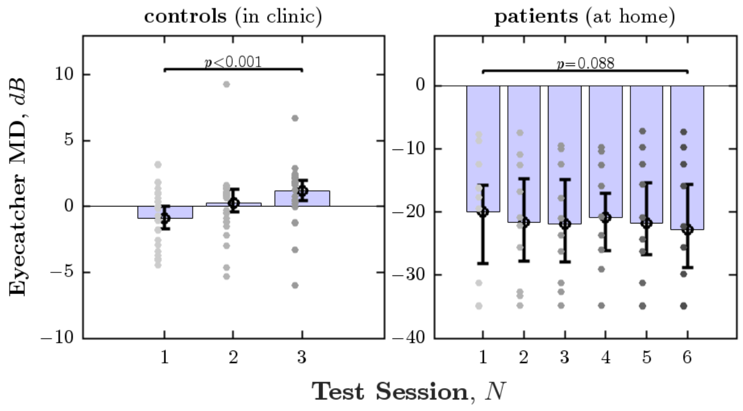

Supplemental Figure S3: Eyecatcher MD as a function of test session for n=16 controls and n=6 patients. The learning effect was significant for controls [$F_{(2, 95)}$ = 13.61, P < 0.001, $\eta^2_{partial}$ = 0.17], but did not reach significance for patients [$F_{(5,59)}$ = 2.06, P = 0.086, $\eta^2_{partial}$ = 0.17], perhaps owing to the small sample size and/or their prior experience with SAP. Note that for simplicity, analyses were crude, One-Way Repeated Measures ANOVAs, assuming independence between the two eyes.

Eyecatcher's System Usability System (SUS) ratings

	Question	Patient				
		IGA006	IGA010	IGA017	IGA003	IGA004
1	I think that I would like to use this system frequently	5	5	4	1	3
2	I found the system unnecessarily complex	1	1	1	4	4
3	I thought the system was easy to use	5	5	5	2	3
4	I think that I would need the support of a technical person to be able to use this system	1	1	1	1	2
5	I found the various functions in this system were well integrated	5	5	4	3	5
6	I thought there was too much inconsistency in this system	2	1	1	3	2
7	I would imagine that most people would learn to use this system very quickly	4	5	5	4	4
8	I found the system very cumbersome to use	1	1	1	5	3
9	I felt very confident using the system	5	5	5	2	5
10	I needed to learn a lot of things before I could get going with this system	2	1	2	1	2
	**Total Score (out of 100):**	92.5	100	92.5	45	67.5

Supplemental Table S1: Breakdown of raw patient responses using the System Usability Scale (SUS). Individual questions are scored from 1 (strongly disagree) to 5 (strongly agree). The total score is computed by subtracting 1 from the odd rows (higher score = better), subtracting the even rows from 5 (lower scores = better), and multiplying the sum total by 2.5.

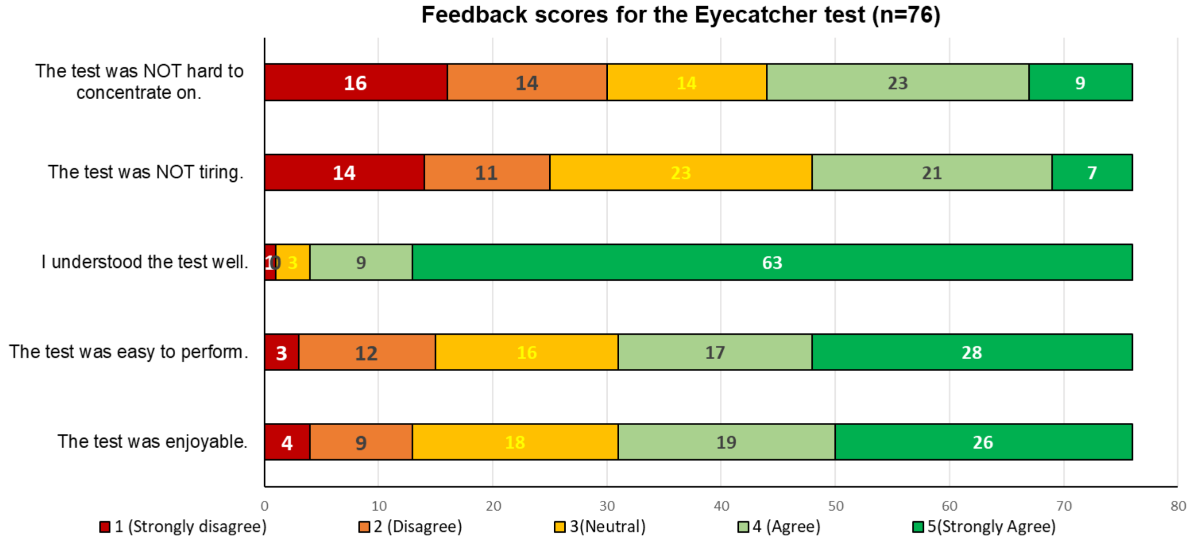

Supplemental Figure S4: Eyecatcher usability ratings from normally sighted young adults. The bars in green shades and red shades represent favorable responses and unfavorable responses respectively. For display purposes only, the first two statements have been inverted ("Not" added to the question and scores reversed) for ease of understanding.
